# Coupling-enabled chirality in terahertz metasurfaces

**DOI:** 10.1515/nanoph-2023-0019

**Published:** 2023-03-01

**Authors:** Shan Yin, Yuting Chen, Baogang Quan, Songyi Liu, Wei Huang, Meng Liu, Wentao Zhang, Jiaguang Han

**Affiliations:** Guangxi Key Laboratory of Optoelectronic Information Processing, School of Optoelectronic Engineering, Guilin University of Electronic Technology, Guilin 541004, China; Beijing National Laboratory for Condensed Matter Physics, Chinese Academy of Sciences, Institute of Physics, Beijing 100190, China; College of Electronic and Information Engineering, Shandong University of Science and Technology, Qingdao 266590, P. R. China; Center for Terahertz Waves and College of Precision Instrument and Optoelectronics Engineering, Tianjin University, Tianjin 300072, China

**Keywords:** chirality, coupled-mode theory, coupling, metasurface

## Abstract

Chirality prevails in nature and is of great value for molecular biology, medicine, and bioscience. Due to the enhancement of chiroptical responses, chiral metasurfaces has attracted enormous attentions. In this paper, some novel polarization-sensitive transmission effects in terahertz chiral metasurfaces are exhibited. In the chiral metasurfaces whose unit cell consists of two basic resonators – a wire and a split ring resonator (SRR), we observe the asymmetrical transmission for circularly polarized state from the circular cross-polarization conversion spectra and the circular conversion dichroism (CCD). More importantly, we verify that the chiroptical activities can be affected by the coupling between the two resonators by simply moving their relative position in the terahertz metasurfaces. From the experimental and simulated results, we observe the distinguished variation in the circular cross-polarization conversion spectra and CCD, and combining with the theoretical analysis using coupled mode theory, we reveal that the chirality of the metasurfaces is strongly correlated to the coupling between the two modes determined by the wire and SRR. Finally, we demonstrate the coupling-enabled chirality by investigating the dependence of CCD on the coupling discrepancy with different relative positions of the two resonators. These findings offer the insights into the relationship between chirality and mode coupling and provide a theoretical method to design chiral metasurfaces and enhance the circular conversion dichroism, which have potential applications in the fields such as optical sensing, polarization imaging, and biological/chemical detection.

## 1 Introduction

Chirality is an intrinsic property of natural materials, manifested as the structure that cannot be coincided with its mirror image by translation or rotation. Chiral objects, such as amino acids, proteins, shells, and even the rotation of planets [1, 2], are widely associated with biomedicine, physics, and chemistry. Due to the peculiar spectral information reflecting the light–matter interactions, chiroptical response, e.g., circular dichroism (CD) measurement, has been a routine for detecting and discriminating chiral matters [3]. However, the subtle chirality of natural molecules increases the challenge in chirality detection, which hinders the relevant researches on the intrinsically physical process.

Recent emerged chiral metamaterials open a new path to explore chiroptical activities. Metamaterials are the artificial subwavelength structures with unnatural properties, such as negative refractive index [4, 5]. By well-arranged nanostructures/microstructures, metamaterials enable manipulating the electromagnetic field, which exhibits the potential applications in optical, microwave, and terahertz devices, like field enhancement [6], polarization converters [7], and super-resolution imaging [8]. Designing asymmetrical structures in metamaterials can usually introduce chirality [9–11]. Compared with natural materials, chiroptical effects induced by chiral metamaterials are orders of magnitude higher [12]. Taking the advantages of flexibility in design, chiral metamaterials have been extensively used in biological monitoring [13], sensing [14–16], detection [9, 17, 18], etc.

Considering the fabrication, the two-dimensional (2D) chiral metamaterials, namely chiral metasurfaces, would be preferred [10], but the chiroptical responses in planar metasurfaces is usually feeble [19]. Therefore, the enhancement of the chiroptical effect has been the hotspot in chiral metasurfaces. Giant circular conversion dichroism (CCD), circular dichroism (CD), and optical rotation (OR) signals have been achieved in bi-layered [20] or multilayered structures [16, 21], and some tunable chiral metasurfaces were reported [22]. However, most works focused on the performance optimization through modifying the geometric constructions. The physical origins of the chiroptical responses in chiral metasurfaces, especially the coupling between the substructures and layers, lack sufficient investigation.

Dealing with the coupling process between different electromagnetic modes, coupled-mode theory (CMT) has been widely used to explain the typical optical and physical phenomenon like electromagnetically induced transparency (EIT) [23], bound states in the continuum (BIC) [24], Fano resonance [25], and so on. CMT can also be applied to describe the mode interactions in chiral metasurfaces [3, 26, 27]. The coupled model derived Born–Kuhn plasma model [28, 29] and biorthogonal approach [3] were proposed to explain the strong chirality in multilayered structures. Besides, the Stokes formula was introduced to characterize the polarization state [29]. However, those structures are usually complicated (3D-shape chiral hole array [26], supercell consisting of eight resonators [27], or multilayered structures [3]), in which the coupling will be intricacy. For example, in the multilayered metasurfaces, the coupling will be affected by many geometrical parameters, such as the shape and relative displacement of the substructures, the distance between the layers, which results in the complexity.

Here, we simplify the chiral metasurfaces to the single metallic array layer deposited on the substrate, which is easy to be fabricated and, more importantly, is uninvolved in the interlayer coupling. The unit cell of the metallic array consists of only two basic resonators – a wire and a split ring resonator (SRR). For simplification, the dimensions of the wire and SRR are fixed to eliminate the impact on their eigen resonances. Hence, via moving the relative position of the wire and SRR, we can straightforwardly investigate the evolution of the chirality resulting from the varying coupling between the two resonators. From the experimental and simulated results, we observe the asymmetrical transmission from the circular cross-polarization conversion spectra and the circular conversion dichroism (CCD), and the distinguished evolution of chirality is derived. Meantime, coupled-mode theory is employed to analyze the chiral spectra, which clarifies that coupling is the vital factor affecting the chirality. According to the dependence of CCD on the coupling discrepancy with different relative positions of the two resonators, the coupling-mediated chirality can be achieved. These results provide a theoretical method to design the chiral metasurfaces with giant circular dichroism or circular conversion dichroism, which may be applied in optical sensing, detection, polarization imaging, and other fields.

## 2 Design and methods

### 2.1 Sample design

**Figure 1:**
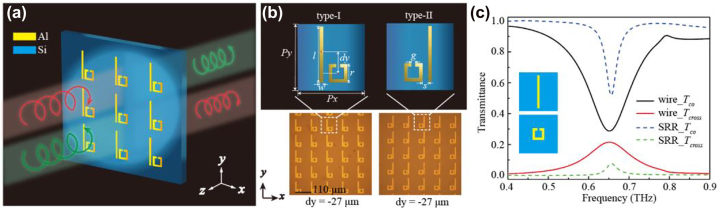
Schematic diagrams and the transmittance of the chiral metasurfaces. (a) Schematic diagram of the chiral metasurfaces. (b) Micrographs of type-I (left) and its mirror structure type-II (right) and their unit cells. (c) Simulated transmittance of bare wire and bare SRR in the unit cell under circularly polarized wave incidence.

The schematic of the chiral metasurfaces is shown in Figure 1(a). A thin layer of 0.2-μm aluminum film is deposited on the silicon substrate with 1 mm thickness. Two groups of mirrored metasurfaces are designed: type-I (SRR sits on the right side) and its enantiomer type-II (SRR sits on the left side) as shown in Figure 1(b), which is used for mutually verifying the coupling phenomenon. The optimized geometric parameters of unit cell structure are as follows: period *P*_*x*_ = *P*_*y*_ = 110 μm, distance between wire and SRR *s* = 5 μm, width of the metallic structures *w* = 5 μm, gap of the SRR *g* = 5 μm, length of the wire *l* = 83 μm, and length of the SRR *r* = 29 μm. We use *dy* to indicate the *y*-axis displacement of the SRR compared to the wire. The period along the *x*- and the *y*-axis is 110 μm to avoid the effect of lattice diffraction [30] at the eigen resonances. Hence, the circularly polarized eigen resonances of the wire and the SRR are tailored to coincide. As shown in Figure 1(c), both structures are excited by the circular polarization and resonant at the nearly same point – 0.652 THz (wire) and 0.656 THz (SRR), thus the strong coupling between them will be induced.

### 2.2 Simulations

Full-wave numerical simulations of the CST microwave studio are used to calculate the chiral spectra of the metasurfaces using the frequency domain solver. The silicon substrate is modeled as a lossless dielectric with permittivity of *ε* = 11.7, while aluminum is set as lossy metal with a conductivity of *σ* = 3.56 × 10^7^ S/m [30]. Periodic boundary conditions are used in the *x*- and *y*-directions, and open boundaries are used in the *z*-direction. Left circular polarization (LCP) (green circle-arrow) or right circular polarization (RCP) (red circle-arrow) wave is normally incident on the metasurfaces. In the simulation, input and output waveguide ports are set in the *z*-direction. Broadband circularly polarized wave is employed as the excitation source, and the circularly polarized transmitted signals are detected.

### 2.3 Circular conversion dichroism

Circular conversion dichroism (CCD) represents the conversion of the incident circularly polarized wave into one of opposite handedness [31–33]. Since the terahertz wave excited from the emitter is linearly polarized, in order to study the response of circular polarization, it is necessary to understand the transmission characteristics between circular polarization and linear polarization. Given the incident plane wave propagation along the *z*-direction, according to the Jones matrix, the circular transmission matrix can be expressed by the linear transmission coefficients as [34, 35]:

(1) $$\begin{array}{ll} t_{\mathrm{circ}} & =\left[\begin{array}{cc} t_{--} & t_{+-}\\ t_{-+} & t_{++} \end{array}\right]\\ & =\left[\begin{array}{cc} t_{xx}+t_{yy}+i\left(t_{xy}-t_{yx}\right) & t_{xx}-t_{yy}+i\left(t_{xy}+t_{yx}\right)\\ t_{xx}-t_{yy}+i\left(t_{xy}+t_{yx}\right) & t_{xx}+t_{yy}-i\left(t_{xy}-t_{yx}\right) \end{array}\right]. \end{array}$$

Here, the subscripts *x* and *y* represent the linearly polarized states, while + and − represent RCP and LCP, respectively, and *t*_*ij*_ represents the transmission coefficient of *i*-polarized transmitted electric field component in response to a *j*-polarized incident electric field of amplitude 1, *i*,*j*∈[*x*,*y*,+,−].

Since the linear cross-polarization conversion is identical in our metasurfaces under normal incidence, namely *t*_*xy*_ = *t*_*yx*_. According to the Jones matrix, the diagonal elements are identical for RCP and LCP waves passing through the structure, which means the circular copolarized conversion *t*_++_ is equal to *t*_−−_ [33]. It means that the metasurfaces have negligible optical activity manifesting in circular dichroism (CD) over the entire spectral range (which can be proved by the simulated and experimental results in the Supplementary material). Consequently, we investigate the chirality by circular conversion dichroism (CCD), which refers to the circular cross-polarization conversion. Hence, the formula of CCD can be written as follows [36]:

(2) $$CCD=T_{+-}-T_{-+}={\left|t_{+-}\right|}^{2}-{\left|t_{-+}\right|}^{2}.$$

where *T*_+−_ or *T*_−+_ represents the transmittance, which is the square of the according transmission coefficient.

### 2.4 Experimental measurement

**Figure 2:**
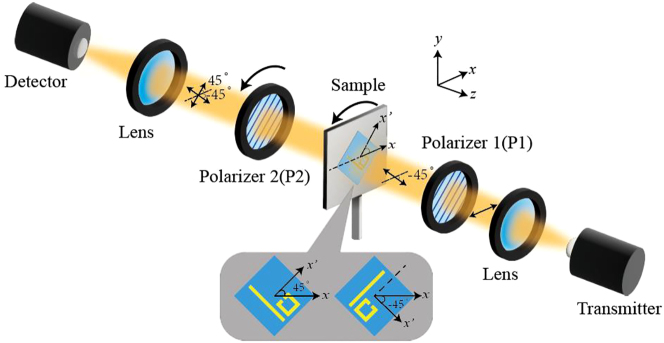
Schematic diagram of the experimental measurement.

The terahertz time-domain spectroscopy (THz-TDS) system is used to measure the transmission spectra of the chiral metasurfaces under normal incidence. According to Eq. (1), we should detect four linearly polarized response signals for a circular polarization component, which is accomplished by a pair of polarizers in the experimental measurement as illustrated in Figure 2. Polarizer 1 (P1) is inserted in front of the sample, and it is rotated by −45° with respect to the incident *x*-polarized wave. Polarizer 2 (P2) is inserted between the sample and the detector. Switching P2 into two orthogonal orientations (parallel or perpendicular to P1), we can obtain the copolarized and cross-polarized conversion transmission, respectively. Coordinate with the rotation of the sample (metasurfaces), which is set to be parallel or perpendicular to P1 as well, we can obtain the four transmission coefficients in linear polarization states (i.e., *t*_*xx*_, *t*_*yy*_, *t*_*xy*_, *t*_*yx*_) required in the Jones matrix, then the complex circular polarization transmission *t*_*circ*_ can be derived (see more details in the Supplementary Information). In addition, the transmission coefficients are obtained by *|t|* = *|E*_*sam*_ *|/|E*_*ref*_ *|*, where the *E*_*sam*_ and *E*_*ref*_ are the measured signal through the sample and reference (bare silicon substrate), respectively.

## 3 Results and discussion

**Figure 3:**
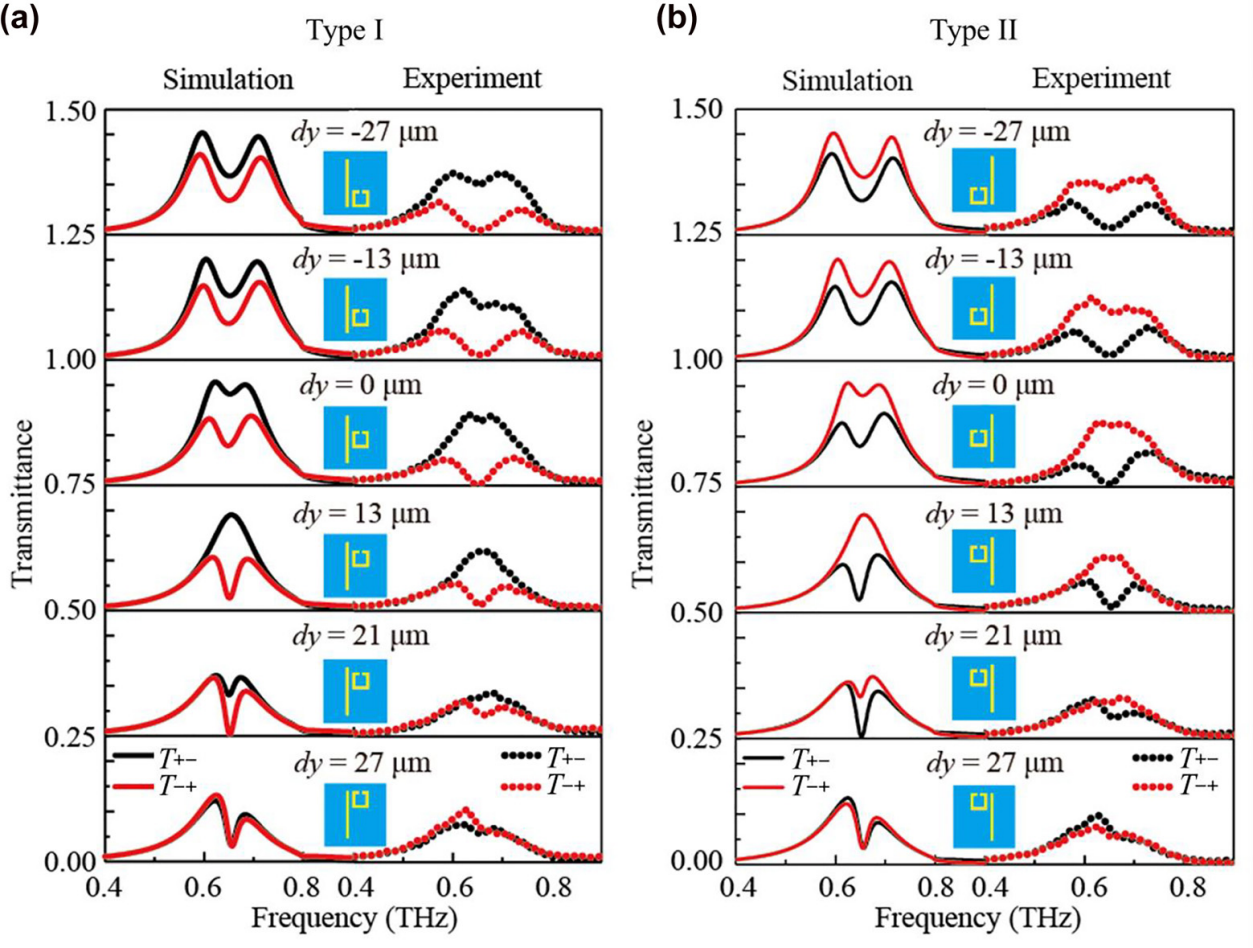
The simulated (solid lines) and experimental (dotted lines) spectra of the metasurfaces for (a) type-I and (b) type-II with varying *dy*. The black and red lines represent the transmittances of *T*_+−_ and *T*_−+_ respectively. For clarification, each spectrum is vertically shifted with 0.25 in sequence.

The transmittance responses of the circular cross-polarization conversion *T*_*ij*_ for the two types of metasurfaces are shown in Figure 3. Here, *T*_+−_ (*T*_−+_) representing the transmittance conversion from RCP to LCP (LCP to RCP) is plotted with the black (red) lines, and the simulated and experimental results are denoted with the solid and dashed lines, respectively. It should be noted that the copolarization transmittances of RCP and LCP are almost the same (see Figure S1 in the Supplementary Information), which means the circular dichroism (CD) can be negligible. Hence, the asymmetrical transmission effect will be embodied in the circular cross-polarization conversion spectra and CCD.

Obvious evolution in the spectra can be seen when the SRR is moved from the bottom to the top (*dy* varies from −27 μm to 27 μm), and the variations of *T*_+−_ and *T*_−+_ are distinguished. For the metasurfaces type-I (Figure 3(a)), the circular transmittance spectra *T*_−+_ show splitting around 0.656 THz, which exactly coincides to the eigen resonance frequencies of the wire and SRR as shown in Figure 1(c). This is the signature of strong coupling [37, 38], which indicates that the coupling occurs between the wire and SRR. When we vertically shift the position of the SRR, the spacing of the two splitting peaks gets narrow as *dy* varies from −27 μm to 27 μm. The circular transmittance spectra *T*_+−_ show the analogous splitting with higher magnitude around 0.656 THz at first (*dy* = −27 μm), and the two splitting peaks gradually approach with the incremental *dy*, however, the splitting vanishes at the case of *dy* = 13 μm; when the SRR is moved up (*dy* is increased) further, the splitting reappears, and the spectrum almost coincides with *T*_−+_ at *dy* = 27 μm. These phenomena certify that the circular polarized response of the metasurfaces can be affected by the coupling between the wire and SRR.

In particular, the reciprocal transmittance spectra of *T*_+−_ and *T*_−+_ are observed in metasurfaces type-II. As in our case, the transmission of type-II can be regarded as the counter propagating transmission of type-I [31]; thus, we can observe the asymmetrical transmission effect by comparing the spectra of type-I and type-II as shown in Figure 3(a) and (b), respectively. Apparently, the chirality responses of type-I and type-II are entirely reciprocal, which is verified by both simulated and experimental data. As a result, the asymmetrical transmission for circularly polarized waves is realized in the planar chiral metasurfaces.

**Figure 4:**
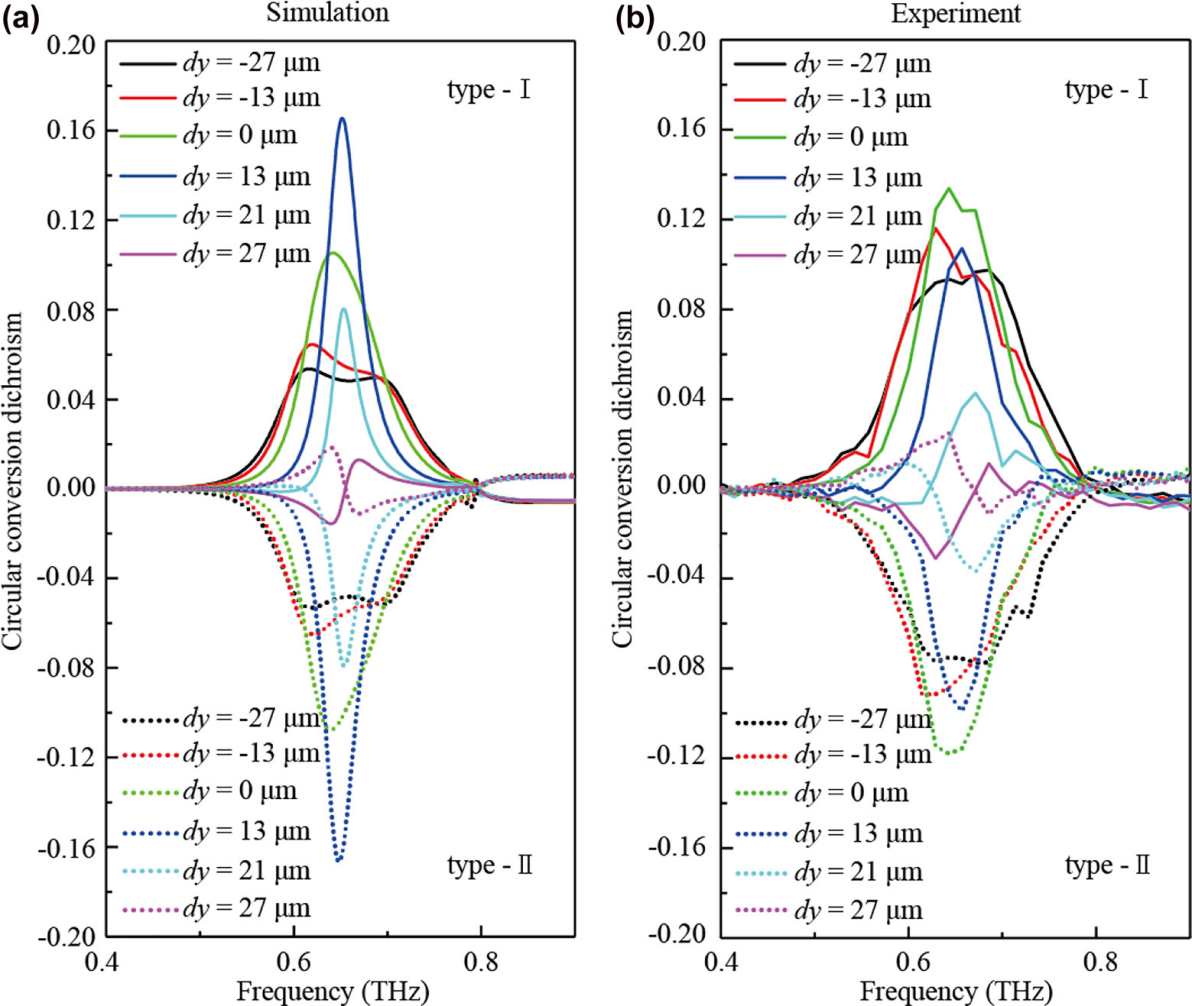
CCD of the chiral metasurfaces. (a) Simulated and (b) experimental CCD spectra of the metasurfaces type-I (solid lines) and type-II (dashed lines) with varying *dy*.

In order to intuitively observe the chirality and the asymmetrical transmission of the metasurfaces with different positional distributions, we calculate the values of circular conversion dichroism (CCD) for clarification. Figure 4(a) and (b) illustrate the simulated and the measured CCD spectra of the two types of metasurfaces, respectively, which are roughly consistent. Here, spectra of type-I and type-II are denoted with solid and dashed lines, respectively. In the simulated results shown in Figure 4(a), the CCD of type I first increases with the incremental *dy* and reaches the maximum value exceeds 0.16 when *dy* = 13 μm, indicating that the chirality reaches the extremum. However, as *dy* further increases, the CCD decreases sharply and finally approaches the minimum nearly zero. For the CCD of type-II, the results are completely reciprocal. The similar variation tendency is also found in the experimental data shown in Figure 4(b), and the reciprocal CCD spectra of type-I and II is evident. It is noted that due to the high sensitivity of the measurement of the four linearly polarized response signals as mentioned in Section 2.4, the errors will be induced if the perfectly parallel or perpendicular situations are not satisfied when we rotate the sample or polarizer 2, which may lead to the CCD maximum presents at different *dy* for the experimental results. The evolution of the CCD demonstrates again that the chiroptical response of the metasurfaces is not only affected by the structural chirality originated from the configurational asymmetry but also involved in the coupling between the resonators.

To reveal how the coupling impact on the chirality, we employ the coupled model theory (CMT) to investigate the chiroptical response of the metasurfaces. CMT is a powerful tool for interpreting the coupling process in metamaterials, which is not only applicable in linear polarization state [39] but also can be utilized in circular polarization state [26, 27]. As we mentioned above, due to the structure optimization, the wire and the SRR can be consistently resonant at 0.656 THz under circularly polarized illumination. And the circular cross-polarization conversion spectra of the wire and the SRR arrays also show resonance peaks accordantly at 0.656 THz as shown in Figure 1(c), and the transmittances *T*_+−_ and *T*_−+_ for each resonator are the same with LCP or RCP excitation. Therefore, the two resonators can be regarded as two bright modes. Hence, we derive the optical response for the circular polarization, which can be described by the following formula [26, 27, 39]:

(3) $$\left[\begin{array}{cc} \omega -\omega_{1}-i\gamma_{1} & g\\ g & \omega -\omega_{2}-i\gamma_{2} \end{array}\right]\left[\begin{array}{c} a\\ b \end{array}\right]=\left[\begin{array}{c} q_{1}E\\ q_{2}E \end{array}\right].$$

Here, all physical parameters are obtained based on the circular polarization case. *ω* is the frequency of the terahertz wave, and *ω*_*1*_(*ω*_*2*_), *a*(*b*), *γ*_*1*_(*γ*_*2*_), and *q*_*1*_(*q*_*2*_) represent the resonance frequency, complex amplitude, loss rate, and the efficiency of external field excitation of the wire (SRR), respectively; *g* is the coupling coefficient between the two modes; and *E* represents the electrical field of the incident terahertz wave. Subsequently, we obtain the energy amplitudes *a* and *b* of each structure by solving Eq. (3):

(4.a) $$a=\frac{\left(\omega -\omega_{2}-i\gamma_{2}\right)q_{1}-gq_{2}}{\left(\omega -\omega_{1}-i\gamma_{1}\right)\left(\omega -\omega_{2}-i\gamma_{2}\right)-g^{2}}E$$

(4.b) $$b=\frac{\left(\omega -\omega_{1}-i\gamma_{1}\right)q_{2}-gq_{1}}{\left(\omega -\omega_{1}-i\gamma_{1}\right)\left(\omega -\omega_{2}-i\gamma_{2}\right)-g^{2}}E$$

Then, we can get the complex transmission coefficient ***t*** as:

(5) $$t=\frac{q_{1}a-q_{2}b}{\varepsilon_{0}E}.$$

And the final transmittance can be derived by

(6) $$T={\left|t\right|}^{2}.$$

**Figure 5:**
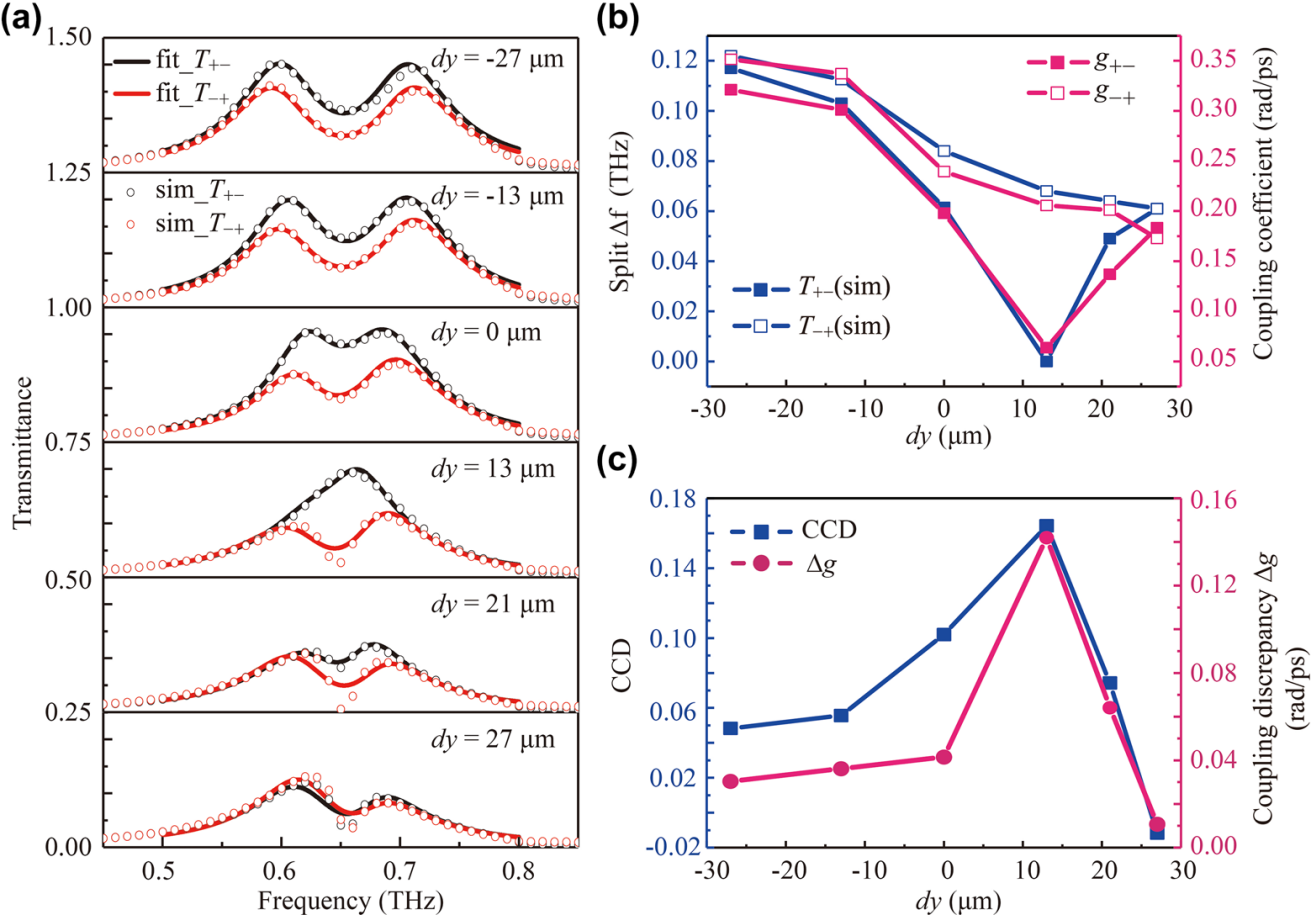
Theoretical analysis results of the coupling enabled chirality. (a) Fitted (solid line) and simulated (open dotted) spectra of the metasurfaces type-I with varying *dy*. (b) Spectral splittings and the coupling coefficients of *T*_+−_ and *T*_−+_. (c) CCD and the coupling discrepancy Δ*g.*

Since the spectra of metasurfaces type-I and type-II are reciprocal as shown in Figure 3, we select the simulated results of type-I to analyze theoretically. Using the Eq. (6), we derive the fitted curves of the circular cross-polarization conversion spectra *T*_+−_ and *T*_−+_, which are plotted with the solid lines in Figure 5(a), and the simulated data are denoted with the open dots. The fitted and simulated results agree well. Then, we extract the key parameter of coupling coefficient *g* by manifesting the coupling strength between the two resonators. The coupling coefficients *g*_+−_ and *g*_+−_ for *T*_+−_ and *T*_−+_ accordingly are illustrated in Figure 5(b) with the blue solid dots and open dots, respectively. It is noted that *g* is a complex number according to Eq. (4), and its real and imaginary parts represent the far field interaction and the near-field coupling between the two resonant modes [39]. From the fitted results, the real part of *g* is much larger than its imaginary part, which means the far-field coupling plays the major role (see Figure S2 in the Supplementary Information). Here, *g* refers to the modulus of the coupling coefficient. Since the spectral splitting (frequency spacing between the two peaks) directly depends on the coupling strength [37, 40, 41], we extract the splitting from the simulated spectra as shown in Figure 5(b) with the red solid dots and open dots, respectively. Obviously, the splittings of the *T*_+−_ and *T*_−+_ vary synchronously with the coupling coefficients, and this agreement convincingly supports our coupled mode theory. As we can see, the strongest coupling occurs at *dy* = −27 μm. As the *dy* increases, the splitting or coupling coefficient of *T*_+−_ monotonically decreases, indicating that the coupling between the two modes is weakened gradually. Whereas, the splitting or coupling coefficient of *T*_−+_ decreases firstly and then increases. The minimum appears at *dy* = 13 μm where the splitting vanishes (i.e., there is only one peak) in the spectrum. These results certify that the chirality of the metasurfaces is strongly correlated to the coupling between the two modes determined by the wire and SRR.

In addition, to intuitively reflect the variation of the chirality, we extract the CCD values at 0.656 THz (the resonance frequency of the two resonators) as a function of *dy*, which are shown in Figure 5(c). We calculate the coupling discrepancy Δ*g* = |*g*_+−_ − *g*_−+_| varying with *dy* as well. The relevance between the coupling and the CCD can be correlated with the transmission spectra. According to our previous work [42], we theoretically illustrated the spectra without coupling can be calculated by Huygens principle; however, as the coupling increases, the deviation of spectra will be enlarged, and the frequency shift of the resonance peak will be distinguished. Thus, the shape change of the spectra will be more severe if the coupling is stronger. Consequently, comparing the coupling discrepancy Δ*g* for *T*_+−_ and *T*_−+_, we could derive the discrepancy of their spectra. As we expected, the CCD changes consistently with the coupling discrepancy. This result further clarifies the positive correlation between chirality and coupling, which can be applied to manipulate the CCD via modulating the coupling between the resonators by simply changing the relative position of the two resonators. Besides, we also fitted the experimental data with CMT, and the similar results and details can be found in the Supplementary Information.

**Figure 6:**
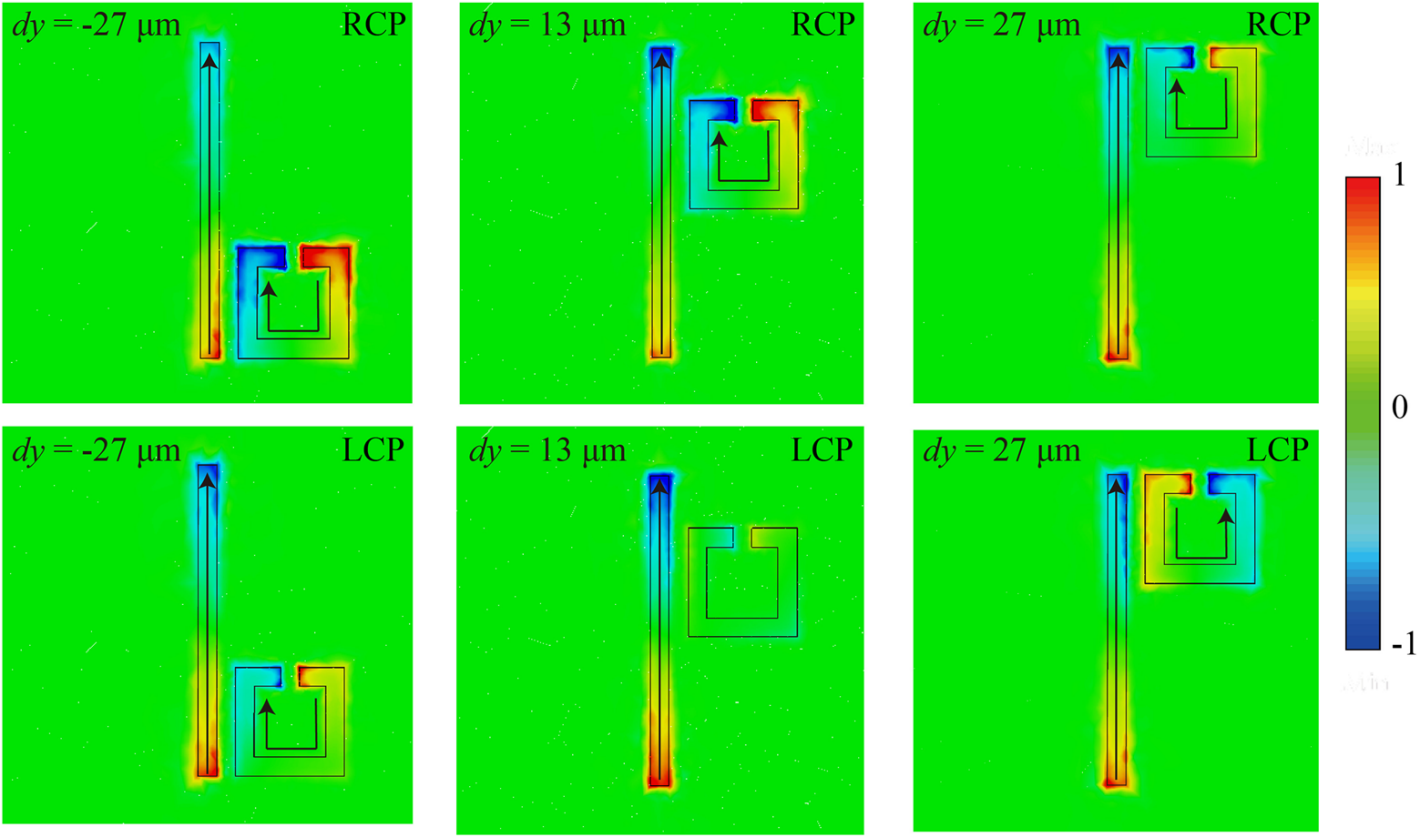
Normalized electric field distributions at different *dy* under RCP (upper panel) and LCP (lower panel) incident waves.

The coupling process also can be investigated via the field distributions. We have plotted the electric field distributions in the *xoy*-plane of the resonance peak excited by the RCP (upper panel) and LCP (lower panel) incident waves, which are shown in Figure 6. As we can see, at most case, both two resonators are excited, which evidences that the two resonators are bright modes. When RCP wave illuminates, the directions of current (denoted with the arrows) for the two resonators remain unchanged. The intensity is strongest at *dy* = −27 μm and decreases with the increase of *dy*, which coincides with the variation tendency of *g*_−+_ in Figure 5(b). When LCP wave illuminates, the electric field under LCP exaction at *dy* = −27 μm is similar to but slightly weaker than that under RCP exaction, which is consistent with the quantitation of coupling strength in Figure 5(b) that *g*_+−_ *< g*_−+_. At *dy* = 13 μm, the SRR is barely excited and corresponds to the weakest coupling of *g*_+−_. When *dy* increases to 27 μm, the currents of SRR turn to the opposite direction, which probably results in the out of phase of the two coupled modes (two resonators) and leads to the smaller coupling strength compared to the situation of in phase at *dy* = −27 *μ*m. Consequently, we support the conclusions of the coupling process via electric field distributions as well.

## 4 Conclusions

We investigate the evolution of the chirality induced by structural asymmetry via moving the relative position of the two resonators in terahertz metasurfaces. And we clarify that the polarization-sensitive transmission effects of the metasurfaces are not only affected by the structural chirality but also involved in the coupling between the resonators. From the experimental and simulated results, we observe the distinguished variation of the chiroptical activities and the asymmetrical transmission through the circular cross-polarization conversion spectra and circular conversion dichroism (CCD), and combining with the theoretical analysis using coupled mode theory, we reveal that the chirality of the metasurfaces is strongly correlated to the coupling between the two modes determined by the wire and SRR. Finally, we demonstrate the modulation of the CCD by changing the relative position of the two resonators. These findings offer the insights into the relationship between chirality and mode coupling and provide a theoretical method to design chiral metasurfaces and enhance the circular conversion dichroism, which have potential applications in optical sensing, polarization imaging, biological/chemical detection, and other fields.

## Supplementary Material

Supplementary Material Details
