# Supplementary material for: Coupling-enabled chirality in terahertz metasurfaces
Source: Nanophotonics. 2023 Mar 1;12(7):1317–26. doi: 10.1515/nanoph-2023-0019 (PMC11636346; doi:10.1515/nanoph-2023-0019)
Supplement: Supplementary file 1 — Supplementary Material Details [file j_nanoph-2023-0019_suppl_001.pdf]

Supplementary Information

Coupling enabled chirality in terahertz metasurfaces

Shan Yin[1], Yuting Chen[1], Baogang Quan[2], Songyi Liu[1], Wei Huang[1]*, Meng Liu[3], Wentao Zhang[1], Jiaguang Han[1,4]**

[1]Guangxi Key Laboratory of Optoelectronic Information Processing,
School of Optoelectronic Engineering, Guilin University of Electronic Technology, Guilin 541004, China
[2]Beijing National Laboratory for Condensed Matter Physics,
Institute of Physics, Chinese Academy of Sciences, Beijing 100190, China.
[3]College of Electronic and Information Engineering,
Shandong University of Science and Technology, Qingdao 266590, P. R. China
[4]Center for Terahertz Waves and College of Precision Instrument and Optoelectronics Engineering, Tianjin University, Tianjin 300072, China
*Email: weihuang@guet.edu.cn
**Email: jiaghan@tju.edu.cn

Supplementary 1: Details about the experimental measurement

Since the terahertz wave excited from the emitter is linearly polarized, according to the Jones matrix (see Eq. (1) in the main body), we should detect four linearly polarized response signals for a circular polarization component. Namely, we must detect t_{xx}, t_{yy}, t_{xy}, t_{yx} first, which is accomplished by a pair of polarizers in the experimental measurement as illustrated in Fig. 2 in the main body.

More specifically, we put the Polarizer 1 (P1) in front of the sample, which is rotated by -45° with respect to the incident x-polarized wave. We first set the sample into the orientation (denoted with x'-axis) parallel to P1, which means that the sample is rotated by -45° with respect to x-axis as well. Next, we set the Polarizer 2 (P2) the orientation parallel to P1 (-45° with respect to x-axis), thus t_{xx} is detected; switch P2 into the orthogonal orientation which is perpendicular to P1 now (45° with respect to x-axis), thus t_{yx} is detected. Similarly, we then rotate the sample by 90° into the orientation perpendicular to P1 (x'-axis is 45° with respect to x-axis), coordinating with the rotation of the P2, which is set to be parallel or perpendicular to P1 as well, we can obtain t_{yy}, t_{xy}, respectively. Hence, the complex circular polarization transmission t_{circ} can be derived according to the Jones matrix Eq. (1).

Supplementary 2: Circular co-polarization spectra

For most chiral metasurfaces, circular dichroism (CD) is important. However, in our proposed metasurfaces as shown in the Fig. 1 of main body, the CD is weak. In this paper, t_{++} indicates the co-polarized conversion from right circular polarization (RCP) to RCP, and t_{--} indicates that from left circular polarization (LCP) to LCP.

Figures S1(a) and S1(b) show the co-polarization transmission spectra t_{++} (green curves) and t_{--} (blue curves) of the metasurfaces type-I and type-II, respectively, and the solid lines and the dotted lines represent the simulated and the experimental result, respectively. Obviously, the simulated transmission spectra of type-I and type-II are the same, which means t_{--} equals to t_{++}. It is well known that CD is defined as the difference between the transmittance of the co-polarization, so the CD of the two metasurfaces is extremely small. It means that the metasurfaces have negligible optical activity manifesting in CD over the entire spectral range. Consequently, we investigate the chirality by circular conversion dichroism (CCD) in the main body, which refers to the circular cross-polarization conversion. It should be noted that there is tiny distinction between t_{++} and t_{--} in the experimental data. But comparing the spectra of type-I and type-II, their trends are the same, i.e., t_{--} is slightly larger than t_{++} in the entire spectral range. It is probably attributed to the limited precision of the sample fabrication.

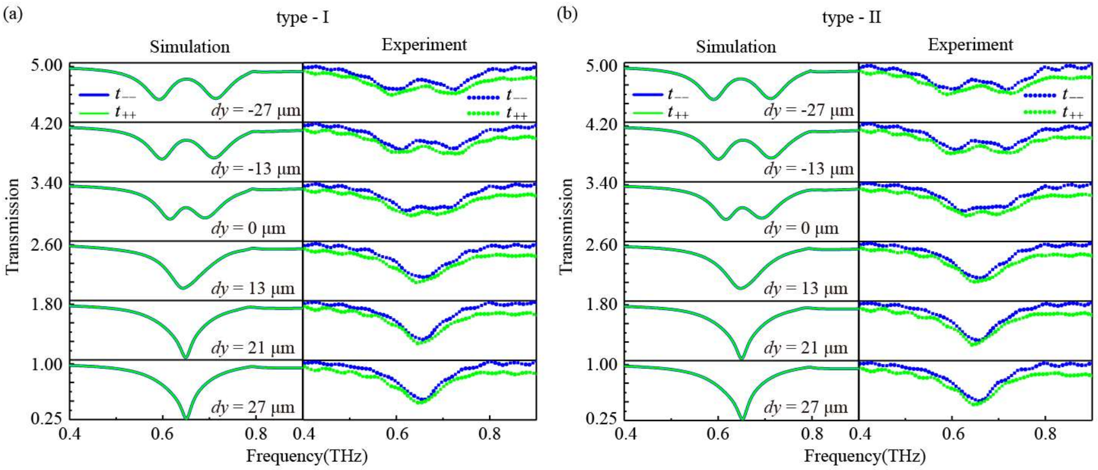

Fig. S1. The simulated (solid lines) and experimental (dotted lines) spectra of the metasurfaces for (a) type I and (b) type II with varying *dy*. The blue and green lines represent the transmission of t_{--} and t_{++}, respectively.

Supplementary 3: Details of the fitting for the simulated results

In this paper, the coupling coefficient g is a complex number according to the Eqs. (4) in the main body. Fig. S2 shows the real (solid dots) and imaginary (hollow dots) parts of the fitted coupling coefficients g_{+-} and g_{+-} of T_{+-} and T_{-+}. The imaginary part of g represents the interaction between the two resonance modes in the near field, while the real part of g represents the far-field coupling between the two resonance modes[1]. As we can see from the fitting results, the real part of g is generally larger than the imaginary part, and in the position with large splitting and strong coupling (the positions except dy = 13 μm), the real part of g is much larger than the imaginary part. As a result, we can conclude that the far-field coupling plays a major role.

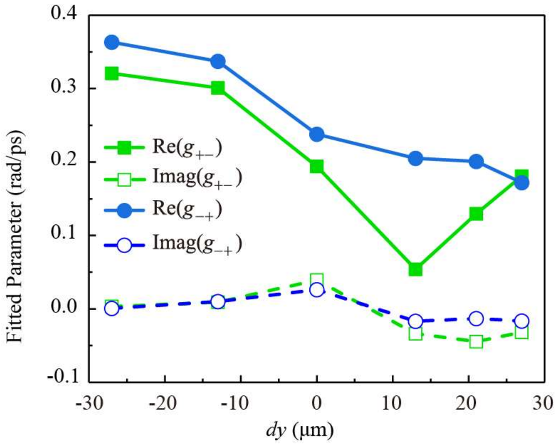

Fig. S2. The coupling coefficients of g_{+-} and g_{-+} as a function of *dy*.

The other fitted parameters with different *dy* are shown in Fig. S3. The solid dots represent the fitted parameters of resonator 1 (wire), and the hollow dots represent the fitted parameters of resonator 2 (SRR). Compared with the variations of the coupling coefficients which can reach 0.3 rad/ps, the variations of the other fitted parameters are much smaller, only about 0.05 rad/ps, so the coupling between two resonators are the main factors influencing the chirality.

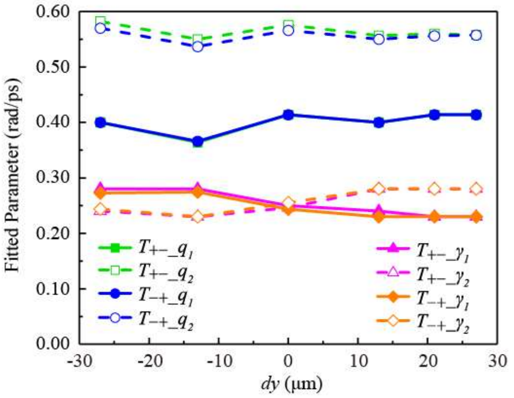

Fig. S3. The fitted parameters of *q* and *γ* as a function of *dy*, while the solid dots represent the parameters of resonator 1 and the hollow dots represent the parameters of resonator 2.

Supplementary 4: Fitting results for the experimental results

We use the CMT to fit the experimental transmission spectra, which are shown in Fig. S4(a). As we can see, the fitted lines (solid lines) agree well with the experimental data are (open dots). And we also extract the key parameter of coupling coefficient *g*. The coupling coefficients g_{+-} and g_{+-} for T_{+-} and T_{-+} accordingly are illustrated in Fig. S4(b) with the blue solid dots and open dots, respectively. Similar to the results for the simulated data shown in Fig. 5 in the main body, the splittings of the T_{+-} and T_{-+} roughly vary synchronously with the coupling coefficients, and this agreement convincingly support our coupled mode theory.

Also, we extract the CCD values for the experimental data at 0.656 THz (the resonance frequency of the two resonators) as a function of *dy*, which are shown in Fig. S4(c). We calculate the coupling discrepancy $\Delta g = |g_{+-} - g_{-+}|$ varying with *dy* as well. It is found that the CCD changes consistently with the coupling discrepancy.

And those fitted results basically coincide with that of the simulated results represented in the main body. The difference is the that in the experimental results, the maximum CCD does not appear at *dy* = 13 μm, which is probably caused by the fabrication errors and the measurement errors.

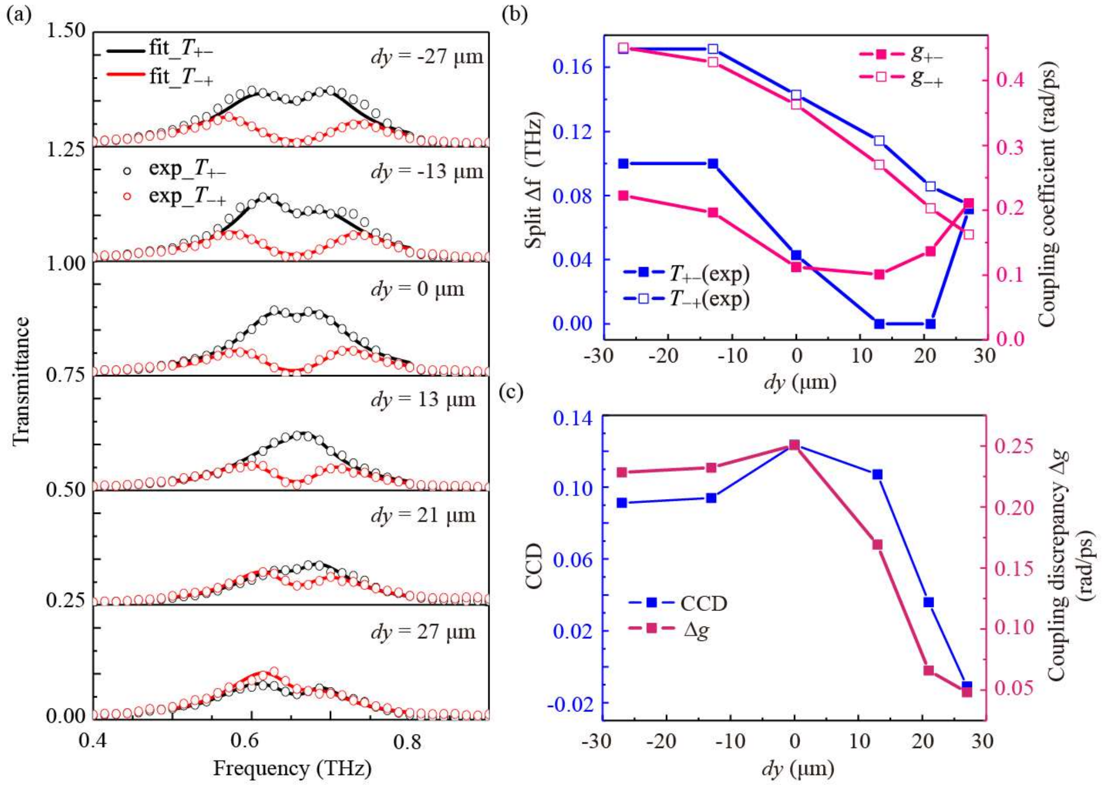

Fig. S4. (a) Fitted (solid line) and experimental (open dotted) spectra of the metasurfaces type-I with varying *dy*. (b) Spectral splittings and the coupling coefficients of T_{+-} and T_{-+}. (c) CCD and the coupling discrepancy Δg.

Reference

[1] L. Niu, Q. Xu, X. Zhang, Z. Zhang, S. Li, X. Chen, Y. Xu, J. Ma, M. Kang, J. Han, and W. Zhang, "Coupling Plasmonic System for Efficient Wavefront Control," *ACS Appl. Mat. Interfaces* **13** (4), 5844-5852 (2021).
